# CD8 T cell response and its released cytokine IFN-γ are necessary for lung alveolar epithelial repair during bacterial pneumonia

**DOI:** 10.3389/fimmu.2023.1268078

**Published:** 2023-10-26

**Authors:** Xiaoying Zhang, Mir Ali, Morgan Alexandra Pantuck, Xiaofeng Yang, Chih-Ru Lin, Karim Bahmed, Beata Kosmider, Ying Tian

**Affiliations:** ^1^ Department of Cardiovascular Sciences, Aging and Cardiovascular Discovery Center, Temple University Lewis Katz School of Medicine, Philadelphia, PA, United States; ^2^ Department of Cardiovascular Sciences, Lemole Center for Integrated Lymphatics and Vascular Research, Temple University Lewis Katz School of Medicine, Philadelphia, PA, United States; ^3^ Department of Microbiology, Immunology and Inflammation, Center for Inflammation and Lung Research, Temple University Lewis Katz School of Medicine, Philadelphia, PA, United States

**Keywords:** CD8 T-cell, IFN-γ, alveolar epithelial cells, repair, acute lung injury

## Abstract

**Introduction:**

Alveolar epithelial regeneration depends on the activity of resident quiescent progenitor cells. Alveolar epithelial type II (AT2) cells are known as the alveolar epithelial progenitor cells. They exit quiescent state, proliferate rapidly in response to injury and differentiate into alveolar epithelial type I (AT1) cells to regenerate the damaged alveolar epithelium. Although AT2 cell plasticity has been a very intense field of research, the role of CD8 T cell response and their released cytokine IFN-γ, in regulating AT2 cell plasticity and alveolar epithelial repair and regeneration after injury remains largely unknown.

**Methods:**

We used flow cytometry to quantify the amount of CD8 T cells in mouse lungs after bacterial pneumonia caused by *Streptococcus pneumoniae*. To determine whether CD8 T cells and their released cytokine IFN-γ are necessary for AT2 cell activity during alveolar epithelial regeneration, we performed loss of function studies using anti-CD8 or anti-IFN-γ monoclonal antibody (mAb) treatment *in vivo*. We assessed the effects of CD8 T cells and cytokine IFN-γ on AT2 cell differentiation capacity using the AT2- CD8 T cell co-culture system *in vitro*.

**Results:**

We detected a transient wave of accumulation of CD8 T cells in mouse lungs, which coincided with the burst of AT2 cell proliferation during alveolar epithelial repair and regeneration in mice following bacterial pneumonia caused by *Streptococcus pneumoniae*. Depletion of CD8 T cells or neutralization of cytokine IFN-γ using anti-CD8 or anti-IFN-γ monoclonal antibody significantly reduced AT2 cell proliferation and differentiation into AT1 cells in mice after bacterial pneumonia. Furthermore, co-culture of CD8 T cells or cytokine IFN-γ with AT2 cells promoted AT2-to-AT1 cell differentiation in both murine and human systems. Conversely, blockade of IFN-γ signaling abrogated the increase in AT2-to-AT1 cell differentiation in the AT2- CD8 T cell co-culture system.

**Discussion:**

Our data demonstrate that CD8 T-cell response and cytokine IFN-γ are necessary for promoting AT2 cell activity during alveolar epithelial repair and regeneration after acute lung injury caused by bacterial pneumonia.

## Introduction

Understanding the effects of immune cells on the regenerative response of lung epithelial progenitor cells to injury is important because immune cells are the largest cell population in the lung and a major target for the treatment of infectious diseases (1, 2). Alveolar epithelial type II (AT2) cells are specialized alveolar epithelial progenitor cells capable of self-renewing and differentiating into alveolar epithelial type I (AT1) cells (3–7). Recent studies have characterized some components of alveolar stroma supporting regenerative niches for AT2 cells (8–12). For example, the monocytes/macrophages have been shown to play an indispensable role in activating AT2 cells and alveolar epithelial regeneration (8, 11), suggesting the importance of innate immune cells in alveolar epithelial regeneration. Nevertheless, our understanding of the roles of adaptive immune system, particularly the contribution of T cells on AT2 cell activity during alveolar epithelial regeneration remains largely unknown.

In adult healthy lungs, there are only a few resident steady-state CD8 T cells, accounting for 1-2% of all CD45+ hematopoietic cells (13). The increase in the number of CD8 T cells and changes in the function of CD8 T cells are usually associated with active recruitment to the inflamed lung during infection (such as bacterial pneumonia) (14). In chronic inflammatory diseases, CD8 T cells are traditionally considered to have harmful effector functions (15). The activity of CD8 T cells causes lung tissue damage by releasing cytotoxic granular cationic proteins stimulated by allergens (16, 17). However, it is increasingly recognized that CD8 T cells have beneficial functions in acute injury environment. For example, CD8 T cells are involved in stem cell activity and repair processes in skeletal muscle regeneration model (18). Despite these important findings, the response of CD8 T cell to acute lung injury has not been well characterized, and the role of CD8 T cell and its secreted cytokine IFN-γ in regulating AT2 cell activity remains unclear.

The purpose of this study was to determine whether CD8 T cell response and their secreted cytokine IFN-γ are necessary for alveolar epithelial repair and regeneration in response to *Streptococcus pneumoniae* (*Sp*)-induced alveolar epithelial injury in mice. In addition, we determined whether CD8 T cells and their secreted cytokine IFN-γ affect AT2 cell activity. To answer these questions, we measured the ability of blocking CD8 T cells or the cytokine IFN-γ to reduce the regenerative response of AT2 cells in mouse lungs. We also measured the ability of CD8 T cells and the cytokine IFN-γ to increase AT2 to AT1 cell differentiation in an *in vitro* AT2 and CD8 T cell co-culture system.

## Materials and methods

### Mice

Mice were bred and raised in the pathogen-free mouse facility at Temple University. C57BL/6, *Rosa26^mTmG^* and IFNγ null mice were purchased from The Jackson Laboratory. Generation and genotyping of the *Sftpc^CreERT2^*; *Rosa26^mTmG^* lines has been previously described (19, 20). These mice were kept on a mixed C57BL/6:129SVJ background. All experiments used 8- to 10-week-old male and female mice and all animal procedures were carried out in accordance with Temple University IACUC.

### Lung inflammation induced by *Streptococcus pneumoniae*

The bacterial infection protocol was executed following previously established methods (19, 21). *Streptococcus pneumoniae* strain T4 (SpT4) underwent cultivation on tryptic soy agar supplemented with catalase (57 μg/ml) under microaerophilic conditions for 14–16 hours at 37°C with 5% CO2. Subsequently, it was subcultured until reaching an optical density of 0.5. The bacterial culture was then subjected to centrifugation, followed by washing with sterile PBS. Just before infection, the bacteria were resuspended in sterile PBS. For the infection procedure, mice aged 8-10 weeks were subjected to intraperitoneal anesthesia using a mixture of ketamine (80-120 mg/kg) and xylazine (5-10 mg/kg). Subsequently, they were intranasally exposed to an inoculum containing roughly 5x10^6 colony-forming units (CFU) in a 30 μl volume of sterile PBS.

### Tamoxifen and 5-ethynyl-2’-deoxyuridine (EdU) administration

Tamoxifen (T5648, Sigma) was dissolved in corn oil (C8267, Sigma) to make a 20 mg/ml stock solution. Mice were given via intraperitoneal injection (200 mg/kg) to activate the Cre recombinase. EdU was administered via intraperitoneal injection (50 mg/kg), followed by a chase of 3 hours.

### Depletion of CD8 T cells and neutralization of cytokine IFN-γ *in vivo*

Mice (8-10 weeks) were administrated with 250 μg of anti-mouse CD8α mAb (clone 53-6.7, Cat# BE0004-1, BioXCell) or anti-mouse IFNγ mAb (clone XMG1.2, Cat# BE0055, BioXCell) or rat IgG2a isotope control (clone 2A3, Cat# BE0089, BioXCell) by daily i.p. injection on days 3-8 after SpT4 infection. On 4 dpi, at 4 hours after last mAb or rat IgG2a treatment, or on 14 dpi, mouse tissues and bronchoalveolar lavage fluid were harvested and processed for flow cytometry and ELISA analysis, respectively.

### Flow cytometry

Mouse lungs and spleens were dissociated according to previously reported procedures (19). Cells were then filtered sequentially through 100- and 40-μm strainers on ice and centrifuged at 200 x g for 10 min at 4°C. Cells were incubated with red blood cell lysis buffer for 1 min on ice and centrifuged, followed by washing with PBS. Cells were resuspended in FACS buffer (1xPBS, 1% BSA, 0.1% NaN3). For live cell staining, dissociated cells were incubated with Fc blocking anti-CD16/32 (Cat# 101320, Biolegend) on ice for 30 min, then with LIVE/DEAD Fixable Aqua Dead Cell Stain (ThermoFisher, #L34966A) along with fluorophore-conjugated antibody mix 1:100 dilution. Cells were then washed twice with FACS buffer and resuspended in FACS buffer. Epithelial cells were selected using the CD45-negative fraction of the cell isolate that stained positively for Ep-CAM (CD326). Within the epithelial cell gate, GFP+, T1α+, or GFP+T1α+ cells were identified and quantified by their geometric mean fluorescence signal intensity. CD8 T cells were selected using CD45+ fraction of the cell isolate that stained positively for CD3 and CD8. CD4 T cells were selected using CD45+ fraction of the cell isolate that stained positively for CD3 and CD4. Neutrophils were selected using CD45+ fraction of the cell isolate that stained positively for Ly6G. Macrophages were selected using CD45+ fraction of the cell isolate that stained positively for CD64. Data was acquired using a BD LSR II flow cytometer and analyzed using FlowJo 10.4.2.

### IFN-γ protein level measurement

Mouse lung bronchoalveolar lavage fluid (BALF) and lung tissue lysates were collected as described previously (21). In brief, BALF was performed with an 800 μl lavages of sterile saline using a 20 G blunt tipped needle inserted into the trachea. Samples were centrifuged at 400 x g for 10 min at 4°C, and the supernatant (BALF) was transferred to a clean tube. The mouse lung tissues were homogenized in PBS containing a protease inhibitor cocktail (MilliporeSigma) and then Triton X-100 was added to a final concentration of 1%. The lysates were frozen at -20°C overnight, thawed on ice, and then centrifuged at 10, 000g for 5 min at 4°C to remove debris. The supernatants were collected and protein concentration was measured using the BCA protein assay. For the AT2 and CD8 T cell co-culture system, culture media from the insert and the well were centrifuged at 400 x g for 10 min at 4°C, and the supernatant (culture media) was transferred to a clean tube. IFN-γ in BALF, lung tissue lysates, and culture media collected above were measured using mouse IFN-γ quantikine ELISA kit (R&D, Catalog #MIF00) according to the manufacturer’s instructions.

### Human lungs

Lungs were obtained from organ donors whose lungs were unsuitable for transplantation and donated for biomedical research through the Gift of Life Donor Program. We selected lungs from donors with a clean bill of lung health history, characterized by a PaO2/FIO2 ratio of >225, no known lung diseases, no signs of infection based on clinical history and X-ray assessments, and limited time spent on a ventilator. A PaO2/FIO2 ration of >225 is indicative of normal or near-normal lung functions, which is essential for efficient oxygen exchange in the lungs. This efficient exchange is crucial for maintaining adequate oxygen levels in the bloodstream and supporting overall health. Our donors were 26-54 years old, including both females and males. AT2 cells were isolated as we previously described (22, 23).

### Antibodies, beads, and chemicals

The following antibodies were used for immunostaining: hamster monoclonal (8.1.1) anti-mouse T1α (1:200, Developmental Studies Hybridoma Bank, University of Iowa), goat polyclonal anti-GFP (1:200, Abcam, ab6673), rabbit monoclonal (SP6) anti-mouse Ki67 (1:100, Abcam, ab16667), chicken anti-goat IgG (H+L) secondary antibody, Alexa Fluor 488 (1:500, Thermo Fisher Scientific, A-21467), goat anti-rabbit IgG (H+L) secondary antibody, Alexa Fluor 568 (1:500, Thermo Fisher Scientific, A-11011), goat anti-hamster IgG (H+L) secondary antibody, Alexa Fluor 568 (1:500, Thermo Fisher Scientific, A-21112). The following antibodies were used for flow cytometry: CD16/32 (Biolegend 101320), CD45/APC-Cy7 (Biolegend 103116), CD3/BV650 (Biolegend 100229), CD4/APC (Biolegend 100412), CD8/PE-Cy7 (Biolegend 100722), CD25/BV421 (Biolegend 102043), EpCaM/BV605 (Biolegend 118227), T1α/BV421 (Biolegend 127423), CD64/APC (Biolegend 139306), Ly6G/BV421 (Biolegend 127628). Anti-human ProSPC (Novus Biologicals, NBP1-60117) and anti-human Ep-CAM (Santa Cruz Biotechnology, 25308) were used for human lung studies. The following antibodies were used for western blotting: mouse monoclonal (E-1) anti-homeodomain only protein HOPX (1:1000, Santa Cruz, sc-398703), mouse monoclonal (C4) anti-gizzard Actin of chicken origin β-Actin (1:1000, Santa Cruz, sc-47778), human anti-RAGE (R&D Systems AF1145) and β-Actin (Sigma A5441). EdU incorporation assay was performed using Click-iT® EdU (5-ethynyl-2’-deoxyuridine) Alexa Fluor® Imaging Kit (ThermoFisher Scientific). T cell activation/expansion kit was purchased from Miltenyi Biotec. Biotinylated anti-CD3 and anti-CD28 antibodies were purchased from eBioscience. Streptavidin-labeled Dynabeads (M280) and CD3/CD28 T cell expander beads were obtained from Invitrogen. Recombinant mouse and human IL-2 and IFN-γ were purchased from PeproTech (Rocky Hill NJ). Anti-mouse and human IFN-γ mAbs and control IgG1 used in cell culture were purchased from BioXcell (anti-mouse IFN-γ mAb: BE0055; anti-human IFN-γ mAb: BE0235; control IgG1: BE0083).

### Preparation of anti-CD3/CD28 beads

To prepare antibody-coated beads of varying composition, streptavidin-labeled beads were coated with varying mixtures of biotinylated anti-CD3 and anti-CD28 antibodies. To this end, streptavidin-M280 beads were washed once with sterile PBS/BSA and resuspended at 10-50 million beads/ml. Preliminary dose response studies, using FITC-labeled anti-mouse IgG and flow cytometry to monitor biotinylated antibody binding to beads, established that beads were saturated by 100 ng of biotinylated antibody/million beads. Consequently, this total immunoglobulin/bead ratio was routinely used for bead coating. To vary the ratio of antibody coating on beads equimolar solutions of anti-CD3 and anti-CD28 were mixed at 1:0, 1:5, 1:10, 1:20, 1:40, 1:80, 2 1:160, and 0:1 ratio. Control beads were coated with biotinylated IgG1 isotype. Coating was performed on a rotator stand at room temperature for 2-3 hours. Beads were then washed two times with filtered PBS/BSA, once with complete medium, and then resuspended in RPMI 1640 complete medium. Antibody coating was performed as needed, but preliminary studies established that beads could be stored 4°C for at least one week without any change in potency. In selected studies, T cells were also stimulated using commercially prepared anti-CD3 and anti-CD28 coated beads (CD3/CD28 T cell Expander, Invitrogen).

### *In vitro* co-culture of mouse AT2 and CD8 T cells

Mouse AT2 cells were isolated following established protocols (19, 21, 24). Briefly, after removing blood from the lungs through PBS perfusion, we introduced dispase (25 U/ml, 37°C) and low melting agarose (1%) into the lungs via the trachea. The agarose was solidified by cooling. Lung lobes were separated, incubated in dispase, minced in DMEM with the addition of DNase I (120 U/ml), and then filtered through strainers. After centrifugation, we used CD45 Microbeads (Cat# 130-052-301, Miltenyi Biotec) to label the cells, followed by purification using MACS LS columns (Cat# 130-042-401, Miltenyi Biotec) on a magnetic separator. The flow-through cells were blocked with FcR blocking reagent (Cat# 130-092-575, Miltenyi Biotec), and subsequently, they underwent incubation with Streptavidin Microbeads (Cat# 130-048-101, Miltenyi Biotec), followed by purification using MACS LS columns. The magnetically labeled AT2 cells were then collected by flushing cold column buffer through the column. Mouse CD8 T cells were purified from splenocytes using mouse CD8α+ T cell isolation kit according to the manufacturers protocol (Miltenyi Biotec, Auburn, CA). Purified CD8 T cells were resuspended in AT2 cell culture medium (DMEM/F12, 1mM L-glutamine, 0.25% bovine serum albumin, 10 mM HEPES, 0.1 mM nonessential amino acids, 0.05% insulin-transferrin-sodium selenite, 100 U/ml penicillin G, 100 μg/ml streptomycin) supplemented with IL-2, without or with IFN-γ, control antibody IgG1, or anti-IFN-γ antibody. Anti-CD3/Anti-CD28 T cell activation beads were prepared according to the manufacturer’s protocol (Miltenyi Biotec, Auburn, CA), and then the beads were added to the T cells in a 1:1 ratio. The mixture of 3 x 10^6 CD8 T cells and anti-CD3/anti-CD28 beads were added to the insert within the well, where they were co-cultured with 1 x 10^6 AT2 cells (**Supplementary Figure 3C**). The co-culture was supplemented with IL-2 (100 units/ml) to prolong the *in vitro* life span of T cells. AT2 cells alone with supplement of anti-CD3/CD28 and IL-2 was used as the control.

### *In vitro* co-culture of human AT2 and CD8 T cells

Isolated human AT2 cells were differentiated to AT1 cells as we reported (22, 23). We isolated CD8 T cells using CD8 Microbeads (Miltenyi Biotec, Auburn, CA) per the manufacturer’s instructions. Co-culture and treatment conditions were applied as described above.

### Western blot

Whole-cell proteins were extracted and western blot was performed as described before (21). Briefly, cells were lysed using RIPA buffer containing protease and phosphatase inhibitors. Protein concentrations were determined using the BCA Protein Assay Reagent kit (BioRad Laboratories). Protein extracts were analyzed on polyacrylamide gels and transferred to nitrocellulose membrane. The blots were blocked in 5% milk in PBST at room temperature (r.t.) for one hour, followed by incubation with primary antibody diluted in blocking buffer at r.t. for one hour. The blots were washed 5 times with PBST and incubated with HRP-conjugated secondary antibodies diluted in blocking buffer at r.t. for one hour, then developed with Thermo Scientific SuperSignal West Femto Maximum Sensitivity ECL substrate, and imaged with GE Healthcare ImageQuant LAS 4000.

### Lung harvest and immunostaining for histology and quantification

Lungs were inflated using the gravity drip and processed for histology as previously described (19, 21). In brief, after perfusing with 10 ml of PBS through the right ventricle, lungs were inflated with 4% paraformaldehyde (PFA) at a pressure of 25 cm H2O pressure gravity drip. The trachea was then tied off and intact lungs were immersed in 4% PFA for 4 hours at 4°C. Lung lobes were separated and washed with cold PBS overnight. After ethanol dehydration, lungs were embedded in paraffin and sectioned at 6 μm. To perform immunohistochemical staining, slides were deparaffinized and rehydrated. Tissue sections were then incubated in citrate buffer (pH6.0) for 20 min at 95-100°C, followed by permeabilization and blocking with 0.3% Triton X-100 and 5% goat serum or horse serum (for goat primary antibodies) in PBS for 1 hour at r.t. Primary antibodies were diluted in PBS and incubated overnight at 4°C. Secondary antibodies were diluted in PBS and incubated for 1 hour at r.t. EdU staining and DAPI nuclear staining were performed according to manufacturer recommendations. Tissue sections were washed 3 x with PBS between antibody incubations for 15 minutes each. Slides were mounted with Aqua-Poly/Mount and images were captured on a Zeiss LSM 710 confocal microscope and a Nikon eclipse fluorescence microscope. Images were processed with ImageJ/FIJI. Quantitation of cell numbers was completed using at least 10 randomly selected regions of each lung per animal.

### Quantitative real time PCR analysis

RNA for real time PCR were extracted using Trizol from cultured mouse AT2 cells. cDNA was produced using random hexamer primers (Cat# N8080127, Invitrogen) and Super- Script III RT (Cat# 18080044, Invitrogen). Real-time PCR was performed using StepOne Plus cycler with SYBR green master mix (Cat# 4367659, Applied Biosystems). Comparative threshold cycle (Delta CT) method was used to transcript expression values and normalized to the expression of *18s* gene. Sequences of the oligonucleotide primers used for qRT-PCR analysis are: *Irf1, forward: 5’-GGGACCCAGCTCTCTTCTGT-3’, reverse: 5’-AAAGCCAGCAAAAGACTCCC-3; Socs1, forward: 5’-GCTGCAGGAGCTGTGTC-3’, reverse: 5’-GGGAAGGAACTCAGGTAGTCA-3; 18s, forward: 5’-TCAAGAACGAAAGTCGGAGG-3’, reverse: 5’-GGACATCTAAGGGCATCAC-3’; IRF1, forward: 5’-CTGTGCGAGTGTACCGGATG-3’, reverse: 5’ -ATCCCCACATGACTTCCTCTT-3’; SOCS1, forward: 5’-CACGCACTTCCGCACATTC-3’, reverse: 5’-TAAGGGCGAAAAAGCAGTTCC-3’; GAPDH, forward: 5’-GGAGCGAGATCCCTCCAAAAT-3’, reverse: 5’-GGCTGTTGTCATACTTCTCATGG-3’.*

### Statistical analysis

Data are reported as means ± standard error of the mean (s.e.m.). Multiple groups were compared by one-way ANOVA followed by Tukey or Dunnett’s *post hoc* test. Two-tailed t test was used when comparing two experimental groups. Multiple groups with multiple time points were compared by two-way ANOVA followed by Sidak multiple comparisons test. A P value less than 0.05 was considered significant. P values were displayed as follows: * P < 0.05; ** P < 0.01; *** P < 0.001; **** P < 0.0001. Results with P > 0.05 were considered not significant (n.s.). All analyses were performed with GraphPad Prism 9.

### Study approval

This study was conducted according to the guidelines outlined by the Public Health Service Policy on the Human Care and Use of Laboratory Animals. All protocols for breeding and experiments with animals were approved by the Institute Animal Care and Use Committee (IACUC), Temple University, protocol number 5012. Studies using human lungs were approved by IRB at Temple University.

## Results

### The level of CD8 T cells is transiently increased in mouse lungs during epithelial repair following SpT4 infection-induced lung injury

**Figure 1:**
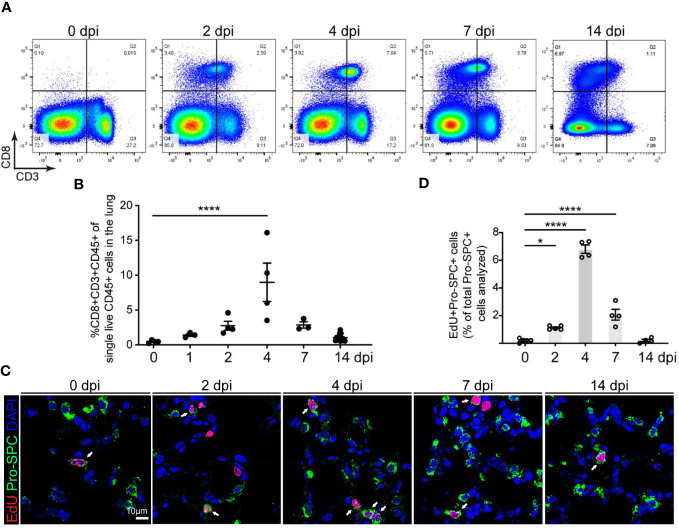
Correlation of CD8 T cell accumulation in the lung and AT2 cell proliferation in SpT4-infected mice. Lung tissues were collected at 0, 1, 2, 4, 7 and 14 days post SpT4 infection (dpi). **(A)** Flow cytometry analysis on dissociated lung cells at 0, 2, 4, 7 and 14 dpi. **(B)** Quantification of flow cytometry data showing the percentage of CD8+CD3+CD45+ cells of total live CD45+ cells in the lung at indicated time points. **(C)** Confocal images of lung sections at 0, 2, 4, 7, and 14 dpi. AT2 cells in DNA synthesis-phase were detected using Click-iT EdU Alexa Fluor (red) and co-immunostaining with antibody against Pro-SPC (green) to detect AT2 cells. Cell nuclear was stained with DAPI (blue). Arrows point to regions double positive for EdU and Pro- SPC. Scale bar: 10 μm. **(D)** Quantification of EdU+Pro-SPC+ cells as percentage of total Pro- SPC+ cells analyzed (≥10 randomly selected fields per mouse). **(B, D)** 3-8 mice per time point. Data are presented as mean ± s.e.m. P values were calculated using one-way ANOVA. * P < 0.05; **** P < 0.0001.

CD8 T cell response induced by pneumococcus infection is the important host defense mechanism during bacterial pneumonia (25). However, the role of CD8 T cells in alveolar epithelial repair and regeneration is largely unknown. We examined the level of CD8 T cells in mouse lungs at various timepoints after *Streptococcus pneumoniae* strain T4 (*SpT4*) infection. Flow cytometry analysis revealed a transient increase in the level of CD8 T cells in mouse lungs after SpT4 infection (**Figures 1A, B**). The percentage of CD8 T cells increased by approximately 18-fold at 4 dpi compared to 0 dpi (8.98 ± 3.24% vs. 0.47 ± 0.11%, 4 dpi vs. 0 dpi, respectively) (**Figure 1B**). It then returned to the basal line by 14 dpi (**Figures 1A, B**). Since the spleen and thymus are the key organs for generating CD8 T cells (26), we next examined their CD8 T cells using flow cytometry analysis. Consistent with the findings in the lung, the transient increase in the level of CD8 T cells was also observed in the spleen and thymus after SpT4 infection. In these tissues, CD8 T cell population increased significantly at 2-7 dpi and returned to the steady state at 14 dpi (**Supplementary Figures 1A, B**).

Our previous studies show that AT2 cells increase their proliferation and differentiation during alveolar epithelial repair and regeneration after SpT4 infection (19). Our 5-ethyl-2’-deoxyuridine (EdU) labeling experiments indicate a transiently increased DNA synthesis in AT2 cells (**Figures 1C, D**). Notably, the peak increase in AT2 cell cycle activity at 4 dpi coincided with the peak increase in the level of CD8 T cells in the lung at this time point (**Figures 1B, D**). We also observed a transient increase in neutrophils in the lung after SpT4 infection (**Supplementary** **Figure 1C**). However, this increase began at 1 dpi and remained high until 4 dpi before CD8 T cell number and epithelial proliferation peaked. These data suggest that SpT4-infected mouse lungs exhibit a transient increase in CD8 T cell level that coincides with AT2 cell cycle activity in mouse lungs during alveolar epithelial repair and regeneration.

### Depletion of CD8 T cells or neutralization of cytokine IFN-γ reduces AT2 cell proliferation after injury

**Figure 2:**
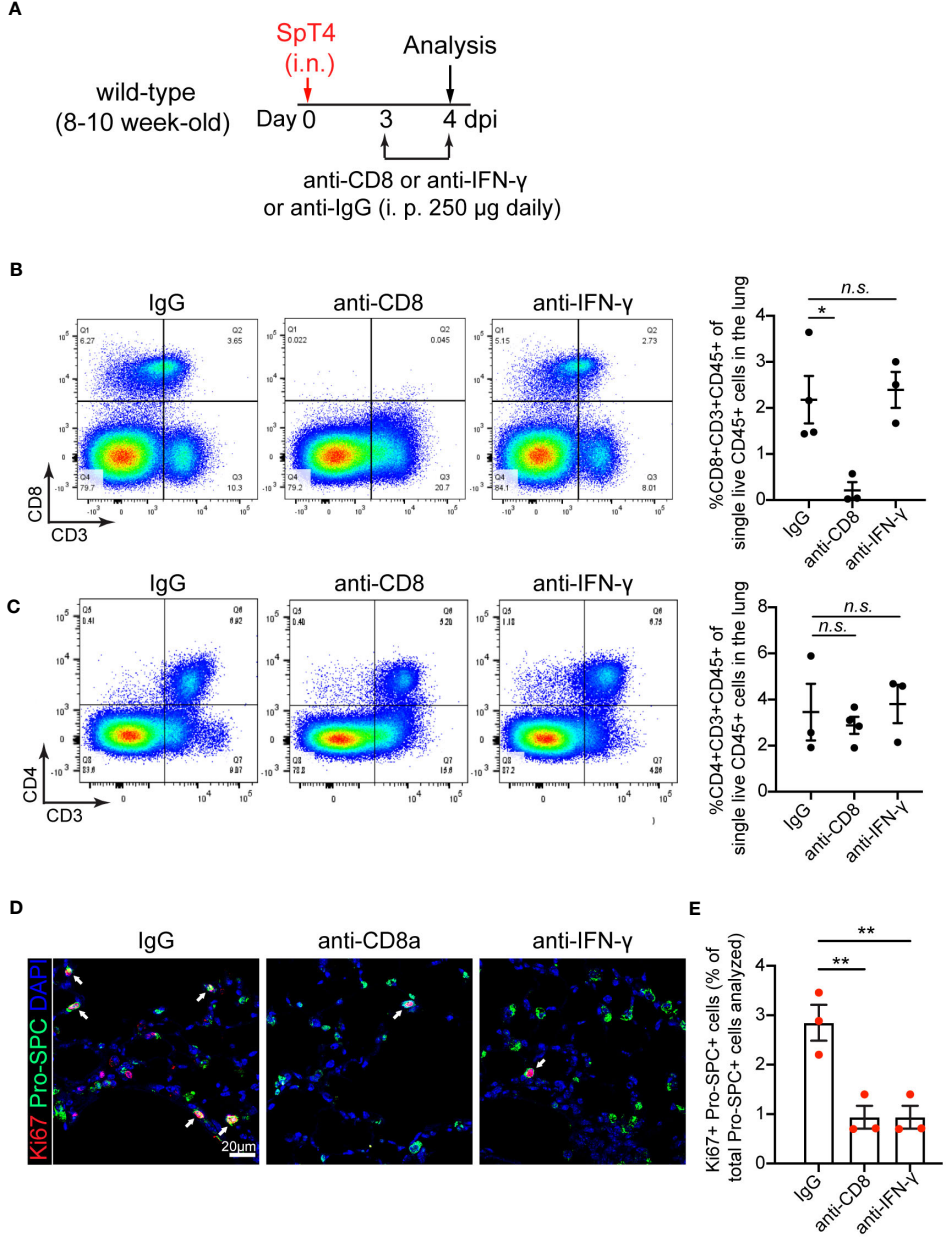
Effect of anti-CD8 T cell and anti-IFNγ treatment on AT2 cell proliferation. **(A)** Schematic of experimental design. **(B)** Flow cytometry analysis on dissociated lung cells showing the percentage of CD8+CD3+CD45+ cells of total live CD45+ cells in the lung at 4dpi. **(C)** Flow cytometry analysis on dissociated lung cells showing the percentage of CD4+CD3+CD45+ cells of total live CD45+ cells in the lung at 4dpi. **(D)** Confocal images of lung sections at 4 dpi. AT2 cells in cell cycle were detected using co-immunostaining with antibody against Ki67 (red) and Pro-SPC (green). Cell nuclear was stained with DAPI (blue). Scale bar: 20 µm. (E) Quantification of Ki67+Pro-SPC+ cells as percentage of total Pro-SPC+ cells analyzed. **(B, C, E)** 3-4 mice per time point. Data are presented as mean ± s.e.m. P values were calculated using one-way ANOVA. * P < 0.05; ** P < 0.01; n.s., not significant.

To determine the contribution of CD8 T cells to AT2 cell cycle activity during alveolar epithelial regeneration, we decreased CD8 T cell levels in SpT4-infected wild-type mice using anti-mouse CD8 antibody, which is commonly used to deplete CD8 T cells without causing T cell activation or inducing an immune response *in vivo* (27–29). Mice were administrated with intraperitoneal (i.p.) injection of anti-CD8 monoclonal antibodies (mAb) (250 μg/day) at 3 dpi and 4 dpi (**Figure 2A**). Of note, anti-CD8 mAb-treated mice revealed a greater than 90% reduction in CD8 T cells in both lungs and spleens, compared with control immunoglobulin G (IgG)-treated mice (**Figure 2B** and **Supplementary Figure 2A**). In contrast, CD4 T cells and macrophages were not significantly affected in mice lungs and spleens with depletion of CD8 T cells (**Figure 2C** and **Supplementary Figures 2B, C**). We next examined AT2 cells proliferation after anti-CD8 mAb treatment. Proliferative AT2 cells were identified by Pro-SPC (AT2 cell marker) staining colocalized with Ki67 (cell proliferation marker). This study revealed that administration of anti-CD8 mAb reduced AT2 cell proliferation, as evidence by the significant decreases in the level of Ki67 and Pro-SPC double-positive cells (Ki67+Pro-SPC+) (**Figures 2D, E**).

It’s known that activated CD8 T cells secrete high amounts of cytokine IFN-γ (30, 31). To determine the contribution of IFN-γ to AT2 cell proliferation, we blocked IFN-γ signaling using anti-mouse IFN-γ mAb. Anti- IFN-γ mAb treatment or anti-CD8 mAb treatment lessened free IFN-γ level in bronchoalveolar lavage fluid (BALF) and lung tissue lysates, verifying the neutralizing efficiency (**Supplementary Figure 2D**). In contrast, anti-IFN-γ mAb treatment did not affect the levels of CD8 T cells, CD4 T cells and macrophages in the lung and spleen, as their levels were similar between anti-IFN-γ mAb-treated and IgG-treated mice (**Figures 2B, C** and **Supplementary Figures 2A–C**). Consistent with outcomes observed in anti-CD8 mAb-treated animals, anti-IFN-γ mAb treatment reduced AT2 cell proliferation after injury (**Figures 2D, E**).

Together, these results indicate that depletion of CD8 T cells and their secreted cytokine IFN-γ inhibited AT2 cell proliferation in response to SpT4 infection-induced lung injury.

### Depletion of CD8 T cells or neutralization of cytokine IFN-γ reduces AT2-to-AT1 cell differentiation and alveolar epithelial regeneration after injury

**Figure 3:**
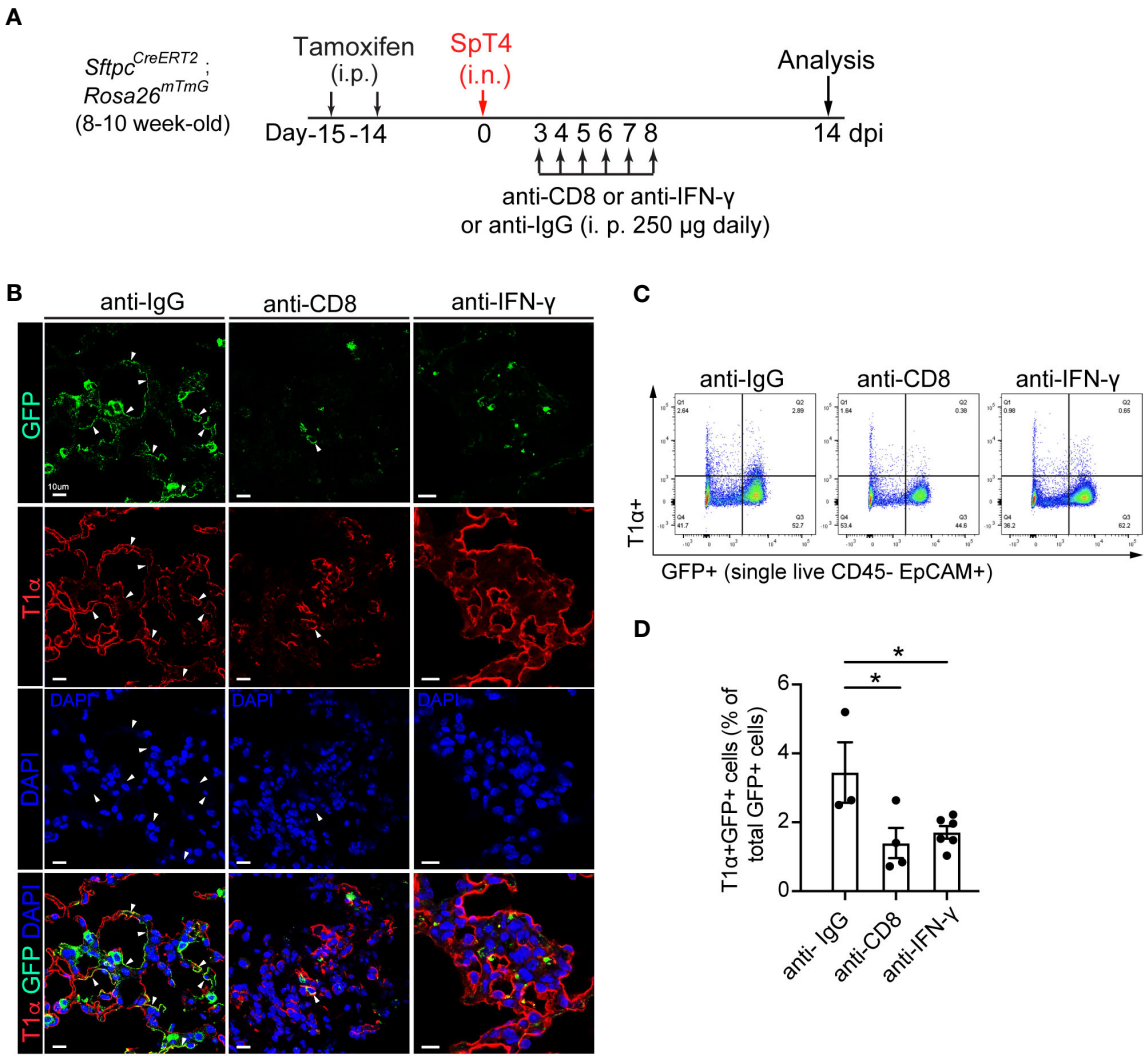
Effect of anti-CD8 T cell and anti-IFNγ treatment on AT2 to AT1 cell differentiation. **(A)** Schematic of experimental design. Mice were administrated with two doses of tamoxifen to label SPC+ AT2 cells. After 14 days of last tamoxifen treatment, mice were infected with SpT4. Mice were i.p. injected with 250 ug of mAb daily between 3 dpi and 8 dpi, and analyzed at 14 dpi. **(B)** Confocal images of lung sections of SftpcCreERT2; Rosa26mTmG mice at 14 dpi. AT2-to-AT1 cell differentiation was visualized by co-immunostaining with antibodies against GFP (lineage-labeled AT2 cells) and T1a (AT1 cell). Arrows pointed to regions doublepositive for GFP and T1α. Scale bar: 10 µm. **(C)** Flow cytometry analysis on dissociated lung cells at 14 dpi. **(D)** Quantification of flow cytometry data showing the percentage of T1α+GFP+ cells of total GFP+ cells at 14 dpi. (D) 3-6 mice per group. P value was calculated using oneway ANOVA. * P < 0.05.

We examined AT2-to-AT1 cell differentiation using a combination of immunohistochemistry and flow cytometry. AT2 reporter mice (*Sftpc^CreERT2^*; *Rosa26^mTmG^*) were administrated with daily intraperitoneal (i.p.) injection of either anti-CD8 mAb or anti- IFN-γ mAb from 3 dpi to 8 dpi (**Figure 3A**). The choice of the 3rd to 8th day for mAb administration was based on our observation of a transient increase in CD8 T cells in the lungs after SpT4 infection (**Figures 1A, B**). Analyses of lung sections through immunostaining and assessing dissociated lung cells via flow cytometry showed that depleting CD8 T cells with anti-CD8 mAb led to a noticeable reduction in AT2-to-AT1 cell differentiation. This reduction was evident in the decreased percentage of lineage-labeled AT1 (GFP+T1α+) cells at 14 dpi compared to control IgG-treated mice (**Figures 3B–D**). Similarly, the neutralization of IFN-γ through anti- IFN-γ mAb treatment also significantly decreased AT2-to-AT1 cell differentiation (**Figures 3B–D**). These results collectively indicate that the depletion of CD8 T cells and their secreted cytokine IFN-γ effectively inhibited AT2-to-AT1 cell differentiation following injury.

### Co-culture of CD8 T cells or cytokine IFN-γ with AT2 cells promotes AT2-to-AT1 cell differentiation *in vitro*

**Figure 4:**
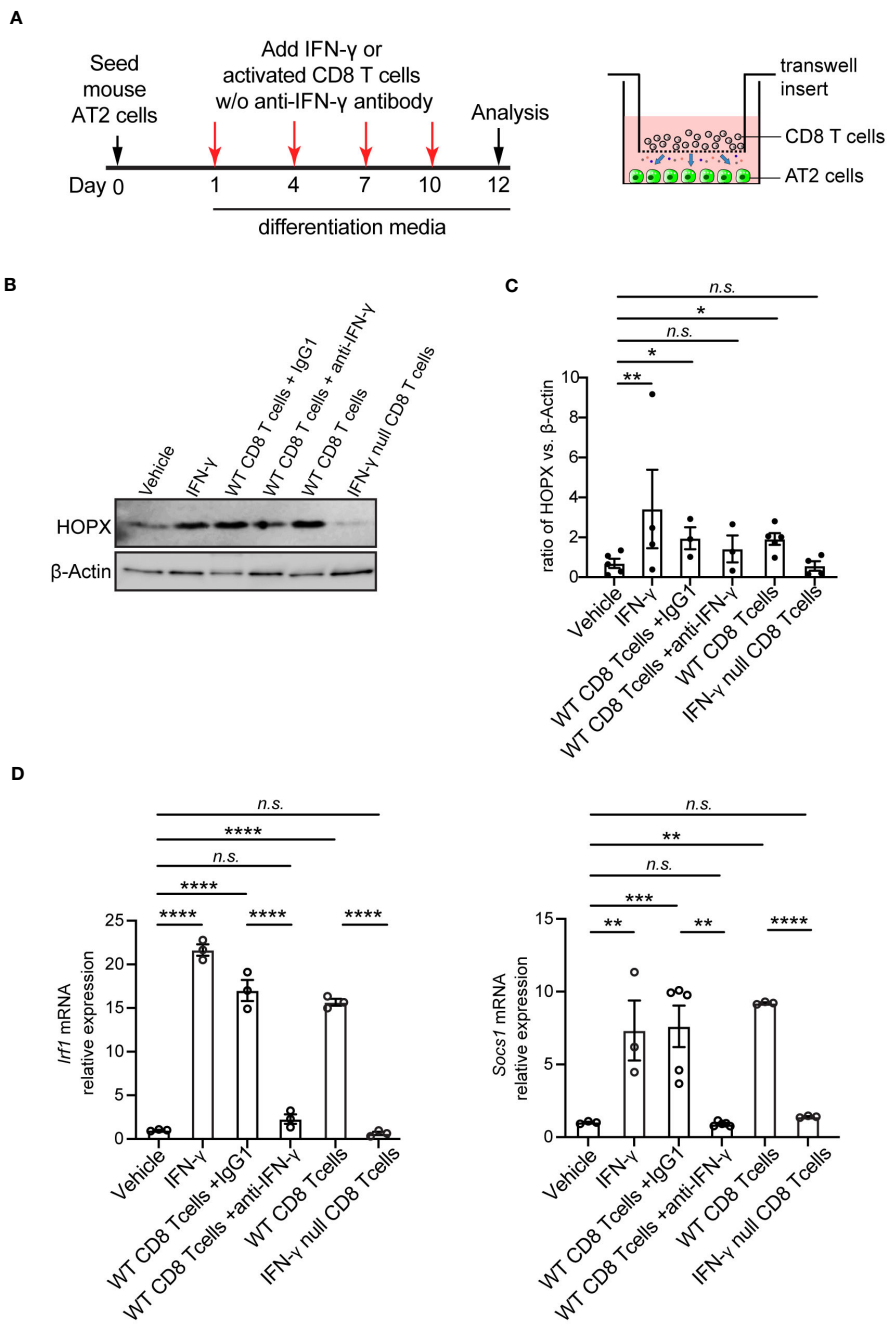
Co-culture of mouse AT2 cells with activated CD8 T cells and cytokine IFN-γ in vitro. **(A)** Schematic of experimental design. **(B)** Western blot analysis (cropped blots) of cell lysates with antibody for HOPX (AT1 cell marker) and β-Actin. **(C)** Quantification of the ratio of HOPX to β -Actin. **(D)** mRNA level of IFN-γ targets, Irf1 and Socs1 on cultured cells using qPCR. Gene expression was normalized to 18s rRNA. **(C)** 3-5 mice per group. **(D)** 3-5 independent experiments. P value was calculated using one-way ANOVA. * P < 0.05; ** P < 0.01; *** P < 0.001; **** P < 0.0001; n.s., not significant.

To address whether CD8 T cells directly influenced the differentiation of AT2 cells into AT1 cells, we utilized an *in vitro* differentiation model of AT2 cells with or without presence of CD8 T cells. AT2 cells were purified from adult wild-type mouse lungs. At 1 and 4 days of culture, CD8 T cells were isolated from adult wild-type mouse spleens and were activated using anti-CD3 and anti-CD28 beads (T cell activation kit, Miltenyi Biotec). Flow symmetry analysis showed that the purity of the isolated CD8 T cells is 95.8% (**Supplementary** **Figure 3A**), and the early activation maker CD25 was increased 2.36-fold on the CD8 T cell surface 24 hours after stimulation with anti-CD3/CD28 beads (1.23% vs. 0.52%, stimulated vs. unstimulated, respectively) (**Supplementary** **Figure 3B**). The activated CD8 T cells were then co-cultured with AT2 cells (**Supplementary** **Figure 3C**). The co-culture was supplemented with IL-2 (100 units/ml) to prolong the *in vitro* life span of T cells (31–33). AT2 cells alone with supplement of anti-CD3/CD28 and IL-2 was used as the control. At 6 days of culture, co-culture of AT2 cells with active CD8 T cells produced a marked increase on AT2-to-AT1 cell differentiation, since the expression of AT1 cell marker HOPX protein was significantly higher in AT2 cell and CD8 T cell co-culture system than in AT2 cell alone system (**Supplementary** **Figure 3D**). To determine whether the stimulatory effects of CD8 T cells on AT2 cell differentiation persist over the long term, we examined AT2-to-AT1 cell differentiation over the course of 12 days of culture (**Figure 4A**). Consistently, co-culture of AT2 cells with active CD8 T cells led to a significant increase on AT2-to-AT1 cell differentiation as evidenced by the higher level of HOPX (AT1 cell marker) protein than the AT2 cell alone system (Vehicle) (**Figures 4B, C**). It has been shown that anti-CD3/CD28 stimulations induce T cells to secrete high amounts of IFN-γ (34, 35). Consistent with this, AT2 cells from co-culture system showed higher levels of mRNAs for *Irf1* and *Socs1*, the downstream target genes of IFN-γ signaling pathway, than the AT2 cell alone system (**Figure 4D**). To determine whether IFN-γ is sufficient to promote AT2-to-AT1 cell differentiation, AT2 cells were cultured and induced to differentiate into AT1 cells with the supplement of mouse IFN-γ. In agreement with the role of activated CD8 T cells in AT2 cell differentiation, adding IFN-γ to the culture medium of AT2 cell promoted the differentiation of AT2 cells into AT1 cells, as evidenced by the significant higher levels of HOPX (AT1 cell marker) protein than the control (Vehicle) (**Figures 4B, C**). To determine whether the promoting effects of CD8 T cells on AT2 cell differentiation and gene expression were mediated through their released cytokine IFN-γ, we blocked IFN-γ in the AT2- CD8 T cell co-culture system using anti- mouse IFN-γ antibody. At 1, 4, 7 and 10 days of culture, anti- IFN-γ antibody (1 μg/ml) was added into the co-culture system. The control group was the co-culture system treated with isotope control IgG1. Treatment with anti- IFN-γ antibody reduced the level of free IFN-γ in the supernatant (**Supplementary** **Figure 3E**). Furthermore, anti- IFN-γ antibody treatment lead to a significant reduction in mRNA levels of IFN-γ signaling target genes (*Irf1*, *Socs1*) (**Figure 4D**),> indicating the blocking effects on IFN-γ pathway. Remarkably, blockade of IFN-γ signaling using anti- mouse IFN-γ antibody abrogated the increase in AT2-to-AT1 cell differentiation in the AT2- CD8 T cell co-culture system (**Figures 4B, C**).

To further determine the role of IFN-γ specifically produced by CD8 T cells, we purified CD8 T cells from adult IFN-γ null mice. CD8 T cells from IFN-γ null mice were activated to the levels even higher than those from wild-type mice using anti-CD3/CD28 beads, as evidenced by the higher frequency of CD25^high^ population (11.6% vs. 1.23%, IFN-γ null vs. Wild-type, respectively) (**Supplementary** **Figure 3B**). Co-culture system of IFN-γ null CD8 T cells and AT2 cells showed near-zero IFN-γ level in the supernatant (**Supplementary** **Figure 3E**.) and no activation of IFN-γ signaling target genes (*Irf1*, *Socs1*) (**Figure 4D**). Consistent with results observed with anti-IFN-γ antibody treatment, blockade IFN-γ signaling using CD8 T cells from IFN-γ null mice abrogated the increase in AT2-to-AT1 cell differentiation in the AT2- CD8 T cell co-culture system (**Figures 4B–D**).

### Human CD8 T cells and cytokine IFN-γ promotes the differentiation of human AT2 cell into AT1 cells *in vitro*

**Figure 5:**
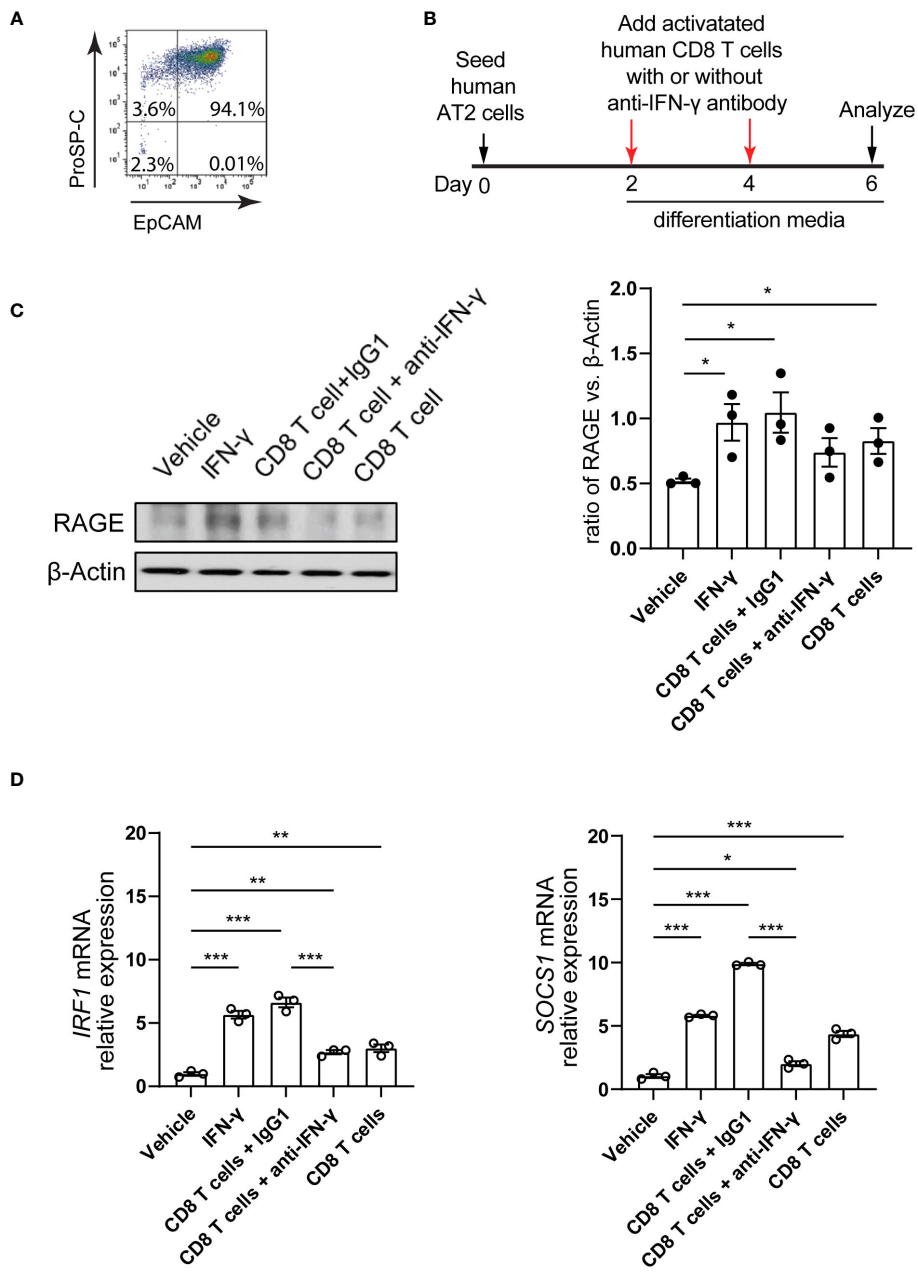
Co-culture of human AT2 cells with activated CD8 T cells and cytokine IFN-γ in vitro. **(A)** Flow cytometry showing purified human AT2 cells. **(B)** Schematic of experimental design. (C) Western blot analysis (cropped blots) of cell lysates with antibody for RAGE (AT1 cell marker) and β-actin. **(C)** Quantification of the ratio of RAGE to β-actin. **(D)** mRNA level of IFN-γ targets, IRF1 and SOCS1 on cultured cells using qPCR. Gene expression was normalized to GAPDH. **(C, D)** 3 independent experiments. P value was calculated using one-way ANOVA. * P < 0.05; ** P < 0.01; *** P < 0.001.

To assess the effects of CD8 T cell response on human AT2 cell differentiation capacity, we isolated human primary AT2 cells and CD8 T cells. Isolated AT2 cells displayed 94% viability and purity (**Figure 5A**). AT2 cells were cultured and induced to differentiate into AT1 cells as we previously described (36, 37). At 2 and 4 days of culture, CD8 T cells isolated from the same human lung were stimulated using anti-human CD3 and anti-human CD28 antibodies, and then were co-cultured with AT2 cells with supplement of human IL-2 (**Figure 5B**). The control condition was AT2 cell alone with supplement of anti-CD3/CD28 and IL-2. Similar to the outcomes observed in our murine system, we found that AT2-to-AT1 cell differentiation was substantially promoted when AT2 cells were co-cultured with CD8 T cells. At 6 days of culture, the co-culture of AT2 cells and CD8 T cells resulted in a significant increase in the expression of RAGE protein (AT1 cell marker) (**Figure 5C**). Furthermore, adding human IFN-γ to the culture medium of AT2 cells increased the expression of the downstream target genes of IFN-γ signaling pathway (*IRF1*, *SOCS1*) and promoted the differentiation of AT2 cells into AT1 cells (**Figures 5C, D**). In contrast, blockade of IFN-γ signaling using anti- human IFN-γ-neutralizing antibody treatment abrogated the increase in AT2-to-AT1 cell differentiation in the AT2- CD8 T cell co-culture system (**Figures 5C, D**).

These results indicate that AT2-to-AT1 cell differentiation was substantially promoted when AT2 cells were co-cultured with CD8 T cells in both murine and human model systems *in vitro*. We identified that CD8 T cells functioned, in part, by its secreted cytokine IFN-γ.

## Discussion

This study in adult mice shows that increased CD8 T cell response after *SpT4* infection is necessary for alveolar epithelial repair and regeneration *in vivo* and that it does so by regulating AT2 cell proliferation and differentiation. Evidence that CD8 T cell response increases is that the amount of CD8 T cells is transiently upregulated in the lungs by *SpT4* infection, and this transient increase in CD8 T cells coincides with the peak increase in AT2 cell cycle activity (**Figure 1D**). The increased CD8 T cell response in the lung appears to be important for AT2 progenitor cell function and alveolar epithelial repair and regeneration. The evidence is that depletion of CD8 T cells using anti-CD8 mAb resulted in decreased AT2 cell proliferation and AT2-to-AT1 cell differentiation after *SpT4* infection (**Figures 2**, **3**). This finding is in accordance with results of studies in skeletal muscle regeneration mice model, which showed that CD8 T cells are necessary for stem cell activity and repair processes (18).

The precise role of CD8 T cells in tissue repair and regeneration remains an active area of investigation, yet the existing evidence remains limited and inclusive. While CD8 T cells are traditionally recognized for their role in immune defense, recent research has suggested potential links between CD8 T cells and tissue regeneration processes, particularly in certain contexts. Nevertheless, it’s essential to acknowledge that the direct evidence demonstrating their regenerative role is currently lacking. Current research efforts have predominantly focused on other immune cell types, including CD4 T cells and regulatory T cells, in the context of tissue repair. Therefore, a deeper understanding of the precise mechanisms by which CD8 T cells may contribute to tissue repair and regeneration requires further research efforts.

The mechanism underlying CD8 T cells’ influence on AT2 cell behaviors appears to be related to the secretion of the cytokine IFN-γ upon CD8 T cell activation. This is corroborated by evidence demonstrating that neutralization of IFN-γ using anti-IFN-γ mAb leads to reduced AT2 cell proliferation and attenuated AT2-to-AT1 cell differentiation after *SpT4* infection, which are similar to the effects observed with anti-CD8 mAb treatment. While some previous studies have posited that CD8 T cell deficiency in the context of skeletal muscle regeneration reduced recruitment of macrophages into muscle, resulting in reduced stem cell numbers (18), our research diverges from this notion. Specifically, our results indicate that depletion of CD8 T cells using anti-CD8 mAb does not significantly affect macrophage levels in the lung. Therefore, we infer that the reduction in cytokine IFN-γ, rather than macrophage activity, is the primary driver of decreased AT2 cell proliferation and differentiation during alveolar epithelial repair and regeneration.

In addition, IFN-γ has been previously reported to play a role in promoting the nuclear translocation and condensation of YAP, thereby augmenting the expression of YAP target genes during anti-PD-1 immunotherapy (38). Previous research, both from our prior work and other research groups, has underscored the importance of YAP nuclear activity in driving AT2 cell proliferation and differentiation (19, 39, 40). Thus, further investigations into the intricate mechanisms through which IFN-γ contributes to lung epithelial repair and regeneration hold the promise of shedding light on these complex processes and potentially informing regenerative medicine approaches.

Interestingly, previous investigations in chronic inflammatory diseases, such as COPD, have suggested that CD8 T cells release cytotoxic granular cationic proteins, which causes lung tissue damage (41). In contrast, our findings reveal that CD8 T cells do not exhibit cytotoxic effects on AT2 cells. Instead, AT2 cells demonstrate an increased capacity for differentiation into AT1 cells when co-cultured with CD8 T cells, observed both in mice and human systems (**Figures 4**, **5**). Furthermore, the increase in AT2-to-AT1 cell differentiation induced by CD8 T cells is abrogated by IFN-γ depletion. The reasons behind this unexpected effect of CD8 T cells on enhancing AT2-to-AT1 cell differentiation are not yet fully understood. One possibility is that the method we used for generating activated CD8 T cells, involving anti-CD3/CD28 beads, may not fully recapitulate the phenotypes of activated CD8 T cells that would naturally arise in lungs with COPD. Additionally, the increased CD25 expression observed on activated CD8 T cells in our study contrasts with previous reports indicating minimal and indifferent CD25 expression on CD8 T cells in both COPD and control (42). This raises the possibility that variations in the phenotype of activated CD8 T cells may contribute to some of the observed cytotoxic effects in COPD.

In summary, our results offer compelling insights into the relationship between CD8 T cell response, IFN-γ secretion, and AT2 cell activity during alveolar epithelial repair and regeneration following SpT4 infection. The increase of CD8 T cell response and IFN-γ secretion coincides with increased activity of AT2 progenitor cells, thereby influencing the process of alveolar epithelial repair and regeneration. Depletion of CD8 T cell or neutralization of IFN-γ results in reduced AT2 cell proliferation and impeded AT2-to-AT1 cell differentiation *in vivo*. Co-culture of AT2 cells with CD8 T cells or cytokine IFN-γ promoted AT2-to-AT1 cell differentiation both in mice and human *in vitro*. These findings, derived from mouse models and human AT2 cell studies, suggest the therapeutic potential of CD8 T cell and IFN-γ in the treatment of infectious lung diseases.

## Data availability statement

The original contributions presented in the study are included in the article/**Supplementary Material**. Further inquiries can be directed to the corresponding author.

## Ethics statement

Studies using human lungs were approved by IRB at Temple University. The studies were conducted in accordance with the local legislation and institutional requirements. The human samples used in this study were gifted from another research group. Written informed consent for participation was not required from the participants or the participants’ legal guardians/next of kin in accordance with the national legislation and institutional requirements. This study was conducted according to the guidelines outlined by the Public Health Service Policy on the Human Care and Use of Laboratory Animals. All protocols for breeding and experiments with animals were approved by the Institute Animal Care and Use Committee (IACUC), Temple University, protocol number 5012. The study was conducted in accordance with the local legislation and institutional requirements.

## Author contributions

XZ: Data curation, Formal Analysis, Investigation, Methodology, Validation, Writing – review & editing. MA: Data curation, Formal Analysis, Investigation, Methodology, Validation, Writing – review & editing. MP: Data curation, Formal Analysis, Investigation, Methodology, Validation, Writing – review & editing. XY: Methodology, Writing – review & editing. C-RL: Data curation, Investigation, Methodology, Writing – review & editing. KB: Data curation, Formal Analysis, Investigation, Methodology, Validation, Writing – review & editing. BK: Supervision, Writing – review & editing. YT: Conceptualization, Funding acquisition, Methodology, Project administration, Supervision, Writing – original draft, Writing – review & editing.
