# Corrigendum: CD8 T cell response and its released cytokine IFN-γ are necessary for lung alveolar epithelial repair during bacterial pneumonia

**DOI:** 10.3389/fimmu.2024.1446350

**Published:** 2024-07-02

**Authors:** Xiaoying Zhang, Mir Ali, Morgan Alexandra Pantuck, Xiaofeng Yang, Chih-Ru Lin, Karim Bahmed, Beata Kosmider, Ying Tian

**Affiliations:** ^1^ Department of Cardiovascular Sciences, Aging and Cardiovascular Discovery Center, Temple University Lewis Katz School of Medicine, Philadelphia, PA, United States; ^2^ Department of Cardiovascular Sciences, Lemole Center for Integrated Lymphatics and Vascular Research, Temple University Lewis Katz School of Medicine, Philadelphia, PA, United States; ^3^ Department of Microbiology, Immunology and Inflammation, Center for Inflammation and Lung Research, Temple University Lewis Katz School of Medicine, Philadelphia, PA, United States

**Keywords:** CD8 T-cell, IFN-γ, alveolar epithelial cells, repair, acute lung injury

In the published article, there was an error in **Figure 1C** as published. In **Figure 1**, “2 dpi” and “7 dpi” were intended to depict separate cells. However, an overlapping region was mistakenly included. The corrected version of **Figure 1** and its caption appear below.

**Figure 1:**
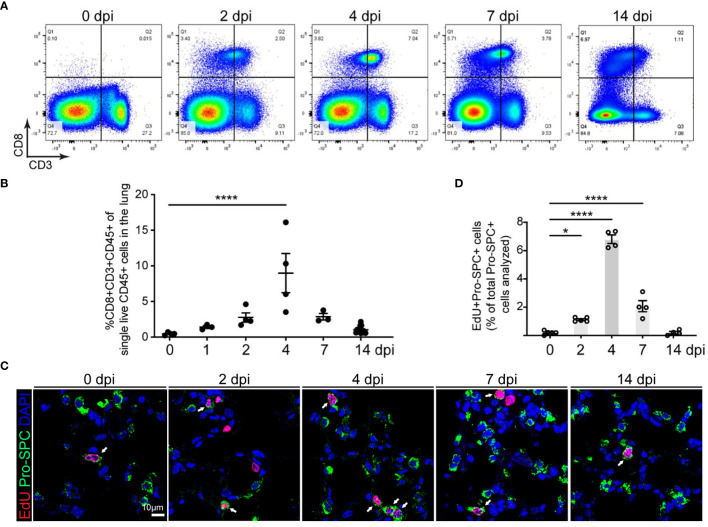
Correlation of CD8 T cell accumulation in the lung and AT2 cell proliferation in SpT4-infected mice. Lung tissues were collected at 0, 1, 2, 4, 7 and 14 days post SpT4 infection (dpi). **(A)** Flow cytometry analysis on dissociated lung cells at 0, 2, 4, 7 and 14 dpi. **(B)** Quantification of flow cytometry data showing the percentage of CD8+CD3+CD45+ cells of total live CD45+ cells in the lung at indicated time points. **(C)** Confocal images of lung sections at 0, 2, 4, 7, and 14 dpi. AT2 cells in DNA synthesis-phase were detected using Click-iT EdU Alexa Fluor (red) and co-immunostaining with antibody against Pro-SPC (green) to detect AT2 cells. Cell nuclear was stained with DAPI (blue). Arrows point to regions double positive for EdU and Pro- SPC. Scale bar: 10 μm. **(D)** Quantification of EdU+Pro-SPC+ cells as percentage of total Pro- SPC+ cells analyzed (≥10 randomly selected fields per mouse). **(B, D)** 3-8 mice per time point. Data are presented as mean ± s.e.m. P values were calculated using one-way ANOVA. * P < 0.05; **** P < 0.0001.

The authors apologize for this error and state that this does not change the scientific conclusions of the article in any way. The original article has been updated.

