# Supplementary material for: CD8 T cell response and its released cytokine IFN-γ are necessary for lung alveolar epithelial repair during bacterial pneumonia
Source: Front Immunol. 2023 Oct 26;14:1268078. doi: 10.3389/fimmu.2023.1268078 (PMC10639165; doi:10.3389/fimmu.2023.1268078)
Supplement: Supplementary file 1 [file DataSheet_1.pdf]

Supplemental Figure 1

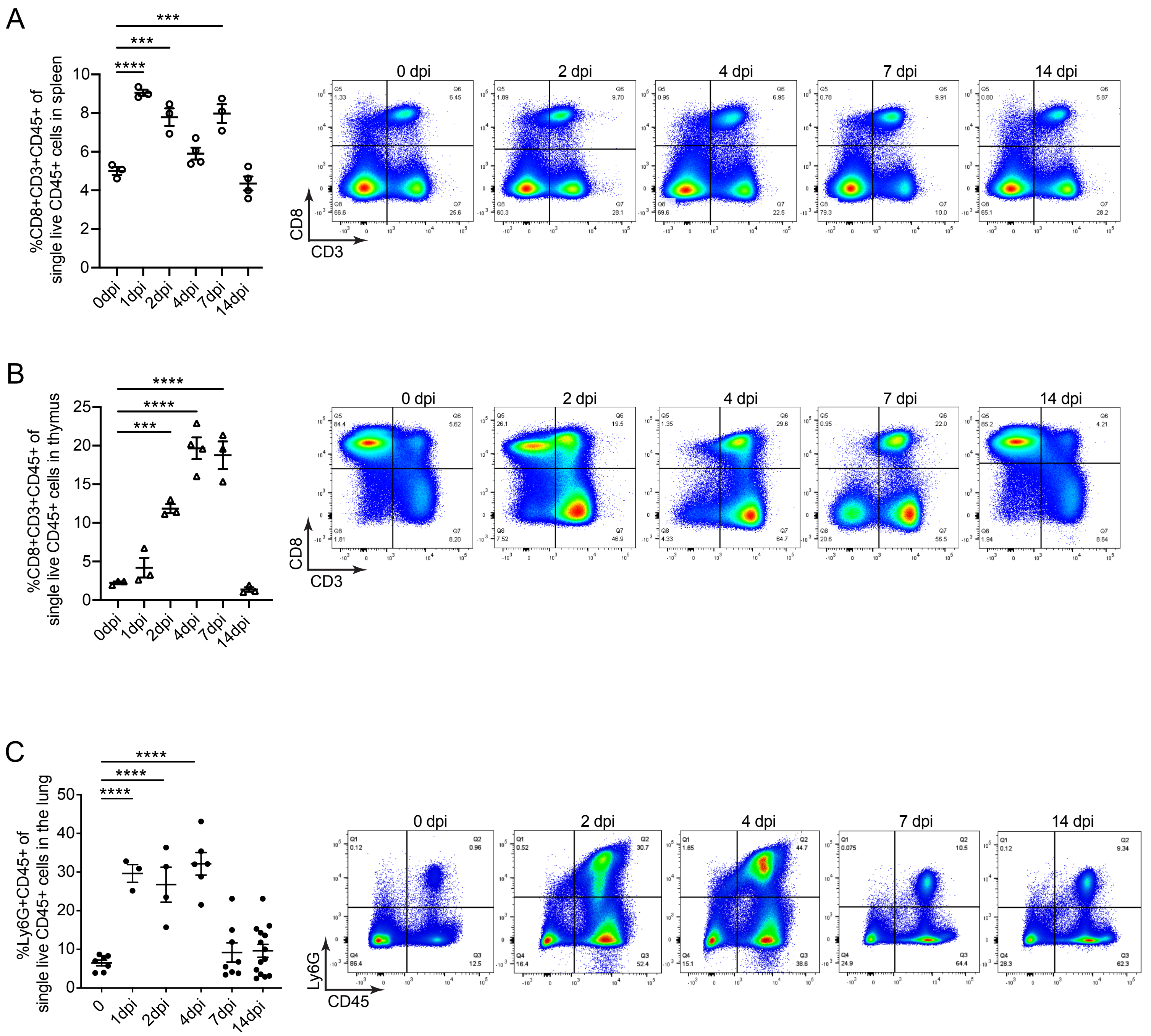

Supplemental Figure 1. Distribution of CD8 T cells and neutrophils in SpT4-infected mice (Related to Figure 1). (A) Flow cytometry analysis on dissociated cells from mouse spleen showing the percentage of CD8+CD3+CD45+ cells of total live CD45+ cells at indicated time points. (B) Flow cytometry analysis on dissociated cells from mouse thymus showing the percentage of CD8+CD3+CD45+ cells of total live CD45+ cells at indicated time points. (C) Flow cytometry analysis on dissociated cells from mouse lungs showing the percentage of Ly6G+CD45+ cells of total live CD45+ cells at indicated time points. (A-C): 3-14 mice per time point. Data are presented as mean ± s.e.m. P values were calculated using one-way ANOVA. *** $P < 0.001$; **** $P < 0.0001$.

Supplemental Figure 2

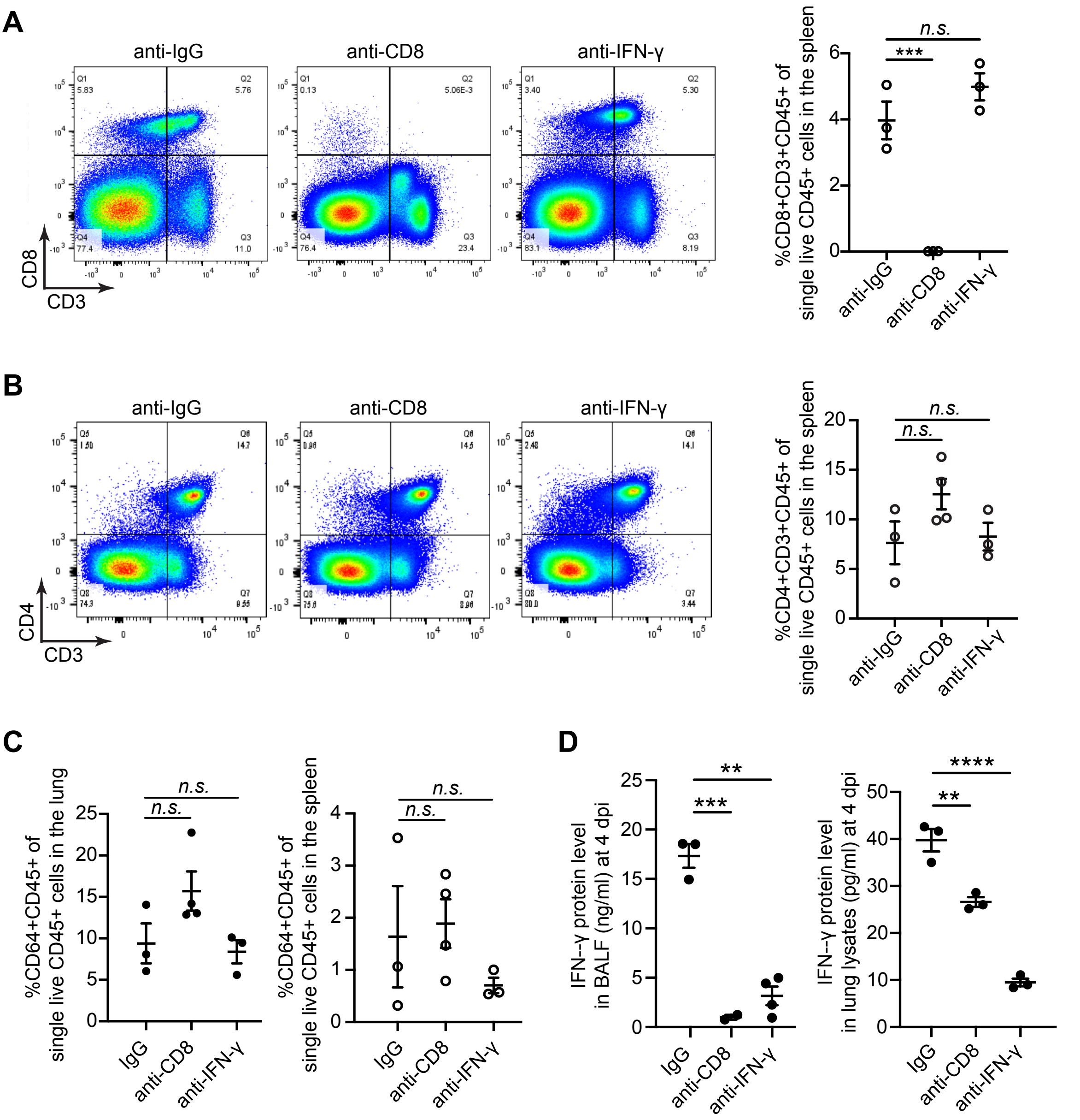

Supplemental Figure 2. Effect of anti-CD8 T cell and anti-IFNγ treatment SpT4-infected mice (Related to Figure 2). (A) Flow cytometry analysis on dissociated cells from mouse spleen showing the percentage of CD8+CD3+CD45+ cells of total live CD45+ cells at 4 dpi. (B) Flow cytometry analysis on dissociated cells from mouse spleen showing the percentage of CD4+CD3+CD45+ cells of total live CD45+ cells at 4 dpi. (C) Flow cytometry analysis on dissociated cells from mouse lungs and spleen showing the percentage of macrophages (CD64+CD3+CD45+) of total live CD45+ cells at 4 dpi. (D) Quantification of IFN-γ protein level in bronchoalveolar lavage fluid (BALF) and lung tissue lysates at 4 dpi using mouse IFN-γ quantikine ELISA kit. (A-D): 3-4 mice per group. P values were calculated using one-way ANOVA. ** $P < 0.01$; *** $P < 0.001$; **** $P < 0.0001$; n.s.: not significant.

Supplemental Figure 3

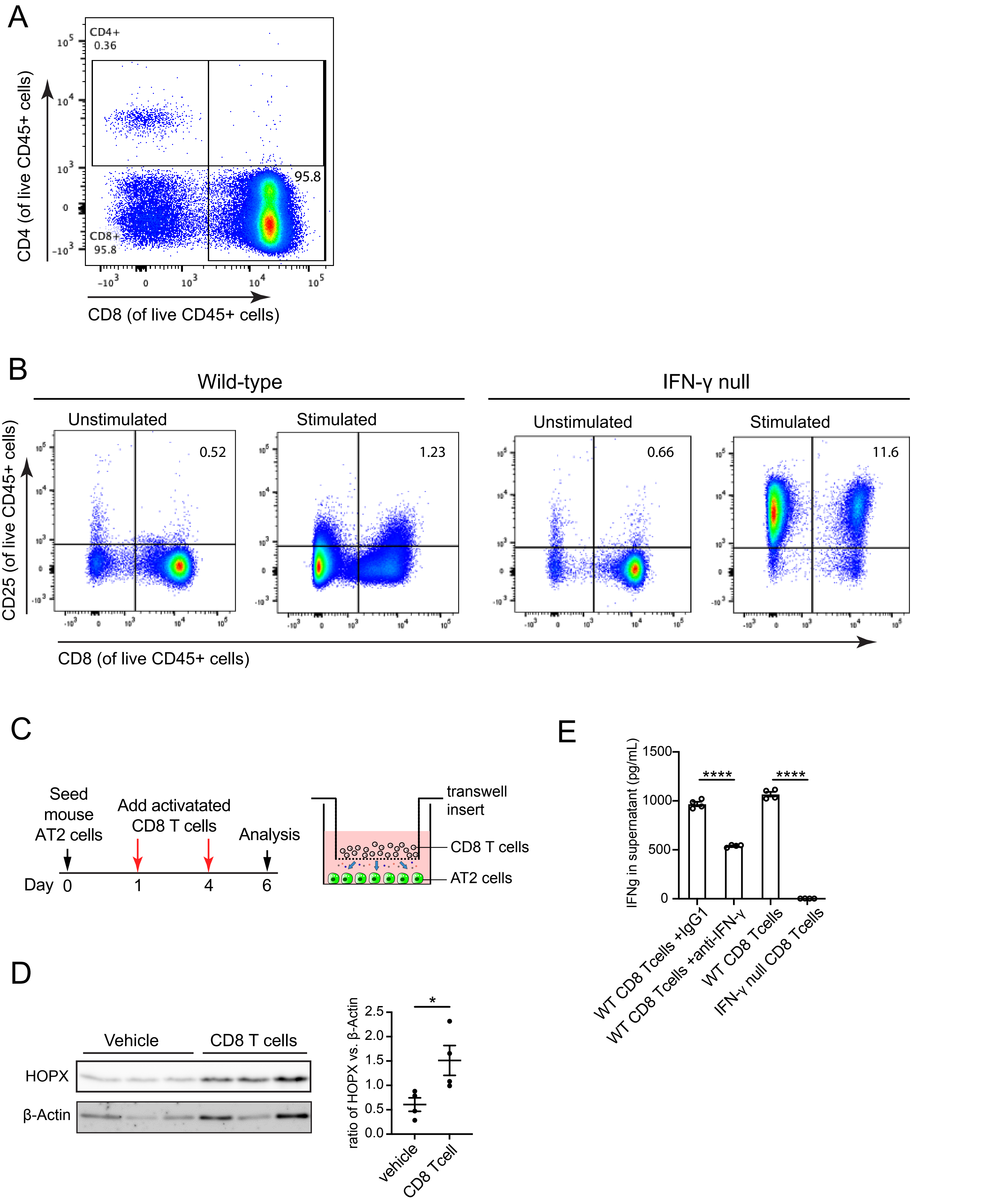

Supplemental Figure 3. Effects of co-culture of CD8 T cell with AT2 cells (Related to Figure 4). (A) Flow cytometry analysis showing purified CD8 T cells from mouse spleen. (B) Flow cytometry analysis showing CD8 T cell activation using the T cell activation/expansion kit. CD8 T cells were purified from the spleen of wild-type or IFN-γ null mice using CD8 T cell isolation kit. Purified CD8 T cells were then activated with anti-CD3/CD28 beads for 24 hrs, and processed for flow cytomery analysis. CD8 T cell activation was identified as CD25+CD8+CD45+ cells. (C) Schematic of experimental design for AT2 and CD8 T cell co-culture system. (D) Western blot analysis (cropped blots) of AT2 cell lysates with antibody for HOPX (AT1 cell marker) and β-Actin. Quantification of the ratio of HOPX to β-Actin was graphed on the right. (E) Protein level of IFN-γ in the supernatant of the co-culture system using ELISA. (D, E): 4 independent experiments. P value was calculated using student t-test (D, E) and one-way ANOVA (G). * $P < 0.05$; **** $P < 0.0001$.
